# Systematic Review of Stem Cells in Plastic, Reconstructive, and Aesthetic Surgery: Clinical Application in Anti-Aging Medicine

**DOI:** 10.1055/s-0045-1812020

**Published:** 2025-10-10

**Authors:** Huntal Napoleon, Putra B. Djohan, Evelyn Evelyn, Laveda Lypinsky, Stefani Luziani, Magistra C. Margaretha

**Affiliations:** 1 Department of Plastic, Reconstruction, and Esthetic Surgery, Indonesia Police Central Bhayangkara Hospital, Jakarta, Indonesia; 2 Department of Medicine, Indonesia Police Central Bhayangkara Hospital, Jakarta, Indonesia; 3 Department of Pharmacy, Indonesia International Institute for Life Sciences, Jakarta, Indonesia; 4 Department of Medicine, Pelita Harapan Faculty of Medicine, Banten, Indonesia; 5 Department of Medicine, Faculty of Medicine, Udayana University, Bali, Indonesia

**Keywords:** stem cells, plastic surgery, anti-aging, facial rejuvenation, wound healing, soft tissue reconstruction, cartilage regeneration, hair restoration

## Abstract

**Background:**

The application of stem cells in plastic, reconstructive, and aesthetic surgery has emerged as a promising frontier in anti-aging medicine. Stem cells, particularly adipose-derived stem cells (ADSCs) and mesenchymal stem cells (MSCs), are increasingly utilized for their regenerative properties, offering innovative solutions for a range of aesthetic and reconstructive challenges.

**Objective:**

This review explores the current and potential clinical applications of stem cells in plastic surgery, focusing on their role in grafting, facial rejuvenation, wound healing, soft tissue reconstruction, and hair restoration.

**Materials and Methods:**

A comprehensive literature analysis was conducted using the PRISMA algorithm, examining studies and clinical trials that evaluate the efficacy, safety, and outcomes of stem cell-based therapies in plastic surgery. Emphasis was placed on the therapeutic mechanisms of stem cells, including their ability to enhance tissue regeneration, reduce scarring, and improve graft survival.

**Results:**

Out of the 5,117 studies identified, 22 met the inclusion criteria, highlighting the diverse applications of stem cells in plastic surgery. These studies reported significant advancements in aesthetic and reconstructive outcomes.

**Conclusion:**

Stem cell therapies represent a transformative approach in plastic surgery, offering significant benefits in aesthetic enhancement and functional restoration. While current applications show promising results, ongoing research is essential to address regulatory issues, ethical considerations, and the need for standardized treatment protocols.

## Introduction

Stem cells have emerged as a groundbreaking medical innovation, offering unique regenerative capabilities that make them highly relevant in plastic, reconstructive, and aesthetic surgery. In anti-aging medicine, the pursuit of rejuvenation and tissue restoration has driven the exploration of cellular therapies to combat the effects of aging, which include decreased skin elasticity, loss of volume, and diminished wound healing capacity.

Among various approaches, stem cells stand out due to their ability to differentiate into multiple cell types and secrete bioactive factors that promote tissue repair and regeneration. Clinical applications, such as fat grafting enhanced by stem cells or the use of mesenchymal stem cells (MSCs) for scar treatment, have shown promising results. Still, significant gaps remain in understanding their long-term safety and efficacy.

This systematic review aims to evaluate the current evidence surrounding the use of stem cells in plastic, reconstructive, and aesthetic surgery, with a particular focus on their role in anti-aging medicine. By synthesizing existing research, we seek to provide insights into their clinical applications, benefits, and limitations, paving the way for future advancements in this field.

## Materials and Methods

### Study Design and Search

This systematic review was conducted in accordance with PRISMA (Preferred Reporting Items for Systematic Reviews and Meta-Analyses) 2020 guidelines to ensure a comprehensive and transparent evaluation of the literature. A systematic search was performed across multiple databases, from inception to November 20, 2024. The following keywords and Medical Subject Headings (MeSH) terms were used: (Stem cells *or* Stem cell therapy *or* Adipose-Derived Stem Cells *or* Mesenchymal Stem Cells) *and* (Plastic Surgery *or* Reconstructive surgical procedures *or* Aesthetic surgery *or* Cosmetic surgery) *and* (Anti-aging medicine *or* Skin rejuvenation *or* Anti-aging therapy *or* Tissue Regeneration). Boolean operators ( *and* / *or* ) and filters (e.g., English-language studies, human subjects) were applied to refine the results.

### Eligibility Criteria

Studies were included if they involved the clinical application of stem cells in plastic, reconstructive, or aesthetic surgery, focusing on anti-aging outcomes such as skin rejuvenation, volume restoration, or scar treatment in the past decade. Eligible studies comprised randomized controlled trials, observational studies, and case series on human samples to ensure robust and clinically relevant findings. Articles were excluded if they lacked original data, such as reviews or editorials, or focused on non-human or preclinical models. Additionally, only studies published in English and Indonesian were considered to maintain consistency and accessibility in data interpretation.

### Data Extraction

The second-fifth reviewer screened titles and abstracts—the second-sixth reviewer reviewed the full text to ensure compliance with the eligibility criteria. The data were then reviewed and discussed with the first reviewer. Discrepancies were resolved by consensus or consultation with the first reviewer. The risk of bias was assessed using appropriate tools, such as the Cochrane Risk of Bias Tool for randomized trials and the Newcastle-Ottawa Scale for observational studies.

To summarize the findings, we compiled a data extraction table ( Tables 1 2 3 4 5 ) detailing the author and year of publication, stem cell type, number of subjects, intervention used, outcome variables studied, and key results. Due to the variability of included studies, data synthesis was performed qualitatively. Extracted information included study design, intervention type, outcome measures, and key findings. A narrative synthesis was used to summarize results, identifying recurring themes in therapeutic mechanisms, clinical outcomes, and advantages of adipose-derived stem cell exosomes (ADSCEs) over adipose-derived stem cells (ADSCs). No direct statistical pooling (meta-analysis) was performed due to heterogeneity in methodologies, outcome measures, and patient populations.

**Table 1:** Stem cell therapy in scar management

Author and year of publication	Stem cell type	No. of subjects ( *n* )	Intervention in study	Outcome variable studied	Result of study
Zhou et al (2016) ^1^	Adipose-derived stem cell-conditioned media (ADSC-CM)	22	ADSC-CM + CO2 laser versus CO _2_ laser only in acne scars on each side of the same face	● Subject satisfaction ● Erythema index ● Melanin index ● Elasticity ● TEWL (transepidermal water loss) ● Hydration ● Skin surface roughness ● Dermal collagen and elastin density	● Satisfaction score higher on ADSC-CM side (2.35 ± 0.69 vs. 2.08 ± 0.76) ● Not statistically significant ● Statistically significant lower average melanin index in the ADSC-CM side ● Statistically significantly higher elasticity in the ADSC-CM side after 1–2 and 3 mo ● TEWL increases after 1 wk and then returns to baseline in both ADSC-CM and CO _2_ only. ADSCM-CM showed significantly lower TEWL ● ADSC-CM side shows a statistically higher hydration score ● Both groups have a significant increase in skin surface roughness ● ADSC-CM side shows significantly higher collagen and elastin density
Kwon et al (2020) ^2^	Adipose-derived stem cell exosomes (ADSCEs)	25	ADSCEs + CO _2_ laser vs. CO _2_ laser only in acne scars on each side of the same face	● ECCA (Échelle D'évaluation Clinique Des Cicatrices D'acné) scores ● 3D image analysis ● Treatment-related erythema	● ECCA shows insignificant differences in the 1st week and later becomes significant in the 2nd and 3rd weeks with ADSCE showing greater reduction ( *p* < 0.01) ● Significant decrease in atrophic scar volume, mean pore volume, and skin surface roughness from baseline on the ADSCE side ● Erythema severity is lower on the ADSCE side ( *p* < 0.03)
Eitta et al (2019) ^3^	Autologous ADSCs	10	Single treatment ADSC vs. 3 sessions of CO _2_ laser therapy for acne scars on each side of the same face	● Scar area percentage ● Skin hydration and TEWL	● Both sides show a significant decrease in scar area percentage after 2 and 3 mo compared with baseline. However, no significant differences were observed between the ADSC and CO _2_ laser side. ● Both sides show a significant improvement in skin hydration and TEWL from baseline without a significant difference between the ADSC and CO _2_ laser side

**Table 2:** Stem cell therapy in wound management

Author and year of publication	Stem cell type	No. of subjects ( *n* )	Intervention in study	Outcome variable studied	Result of study
Park et al (2024) ^4^	Pharyngeal-derived exosome	5	Case report of exosome application in wound healing	● Case 1: nasal ischemic necrosis due to a polycaprolactone filler. ● Case 2: abrasive wound in the immature scar stage ● Case 3: third-degree burn injury (contact burn) ● Case 4: mature keloid treated with exosomes and micro-needling ● Case 5: flap necrosis prevention in facelift	● Total wound closure and epithelization within 3 mo of application ● Notably promoted wound regeneration and reduced keloid formation ● Complete wound closure and mature scar formation within 3 mo of application ● Beneficial effects in treating mature keloids ● Prevented complete necrosis and effectively promoted wound healing
Kim et al (2016) ^5^	ADSCs	12	ADSC application in complicated wounds following filler injections	● Vancouver scar scale	● The study states the improvement of the Vancouver scar scale without further statistical analysis

**Table 3:** Stem cell therapy in burn management

Author and year of publication	Stem cell type	No. of subjects ( *n* )	Intervention in study	Outcome variable studied	Result of study
Joo et al (2023) ^6^	Human stem cell-conditioned medium (HSCM)	14	Non-ablative laser + HSCM on burn-induced hypertrophic scar	● Scar thickness ● Erythema ● TEWL ● Cutometer parameters	● Significant differences were observed between the pre- and posttreatment measurements of erythema ( *p* < 0.001), ● TEWL ( *p* < 0.001), and ● Cutometer parameters (all parameters; *p* < 0.05) of experimental scars

**Table 4:** Stem cell therapy in skin rejuvenation

Author and year of publication	Stem cell type	No. of subjects ( *n* )	Intervention in study	Outcome variable studied	Result of study
Zhou et al (2016) ^7^	Autologous bone marrow mononuclear cell (MNC)	38	Skin expansion (mechanical stretch) received intradermal injections of MNCs or a placebo	● Expansion index (EI) ● Skin surface area ● Skin thickness ● Skin texture ● Histological indicators: cell proliferation (PCNA ^+^ ) and angiogenesis (CD31 + ) ● Safety	● EI significantly higher in MNC group at 4 and 8 wk ( *p* = 0.001) ● Skin surface area, skin thickness, skin texture, proliferating cells, and angiogenesis were significantly higher in the MNC group ( *p* < 0.001) ● There are no serious adverse events reported, including at 2-y follow-up
Purwati et al (2020) 8	Mesenchymal stem cells (MSCs) derived from the placenta	30	Topical application of a stem cell metabolites formula derived from human placenta. Applied twice daily (morning and night) for 6 months on the face	Using Janus Skin Analyzer, including: ● Spot ● Pore ● Roughness ● Wrinkle ● UV acne ● UV spot ● UV moisture	● Spots, pores, roughness, wrinkles, UV acne, and UV spots showed a significant decrease over 6 mo ● UV moisture significantly increased, indicating improved skin hydration ● 90% of subjects were satisfied (reporting smoother skin, reduced wrinkles, and improved pigmentation) ● All changes were statistically significant ( *p* < 0.05)
Gyeon-Hun Park et al (2023) ^9^	HACS (human adipose tissue stem cell-derived exosome-containing solution)	28	Application of human adipose tissue stem cell-derived exosome-containing solution in conjunction with microneedling	● Wrinkle improvement ● Skin elasticity ● Skin density ● Patient satisfaction	● Wrinkle severity significantly decreases in WSRS scores on the treated side compared with the control side ● Skin elasticity and skin density improvement on the treated side ● Patient satisfaction was reported to be higher on the treated side
Wang et al (2018) 10	Adipose-derived stem cell (ADSC)-conditioned medium (CM)	30	Application of protein extracts from the medium of ADSCs using microneedles	● Melanin index ● Skin color (ITA° value) ● Skin gloss/radiance ● Skin surface topography (smoothness, roughness, wrinkles, scaliness) ● Skin elasticity ● Periorbital skin relief ● Self-evaluation questionnaire (wrinkles, firmness, elasticity, hydration, whitening, radiance) ● Safety assessment	● Melanin index and wrinkle significantly decrease on the test side ● Skin brightness, skin radiance, and skin elasticity significantly increase on the test side than the control ● Skin surface topography improved, but the AAPE side showed better overall texture ● Patient satisfaction: over 70% of participants reported higher ● No adverse effects observed, and the treatment was well-tolerated
Svolacchia et al (2024) 33	ADSC-derived exosomes	72	Application of the derived exosomes and nanovesicles to targeted skin areas	● Skin regeneration ● Antiaging effects ● Safety and tolerability	● Significant improvements were observed in patient satisfaction scores on the NRS and Berardesca scale, with statistical significance ( *p* < 0.0001)
Lee et al (2014) ^11^	Human embryonic stem cell-derived endothelial precursor cell (hESC-EPC) conditioned media (CM)	25	Treating one side of each participant's face with microneedling combined with hESC-EPC CM treatments was administered in five sessions at 2-wk intervals	● Pigmentation (melanin index) ● Erythema (redness index) ● Wrinkle depth and roughness ● Photographic assessment ● Participant satisfaction	● Pigmentation and erythema significantly decrease on the hESC-EPC CM side ( *p* < 0.05) ● Significant improvements in wrinkles on the hESC-EPC CM side ( *p* < 0.05). ● Greater improvement in pigmentation and wrinkles was observed on the hESC-EPC CM-treated side ● Higher satisfaction score with hESC-EPC CM treatment (3.25 ± 1.26) compared with control (2.72 ± 1.45) ( *p* < 0.05)

**Table:** 

Young in Lee et al (2020) ^12^	adipocyte-derived mesenchymal stem cell-conditioned medium (ADSC-CM)	25	One side of the face was treated with a cream containing ADSC-CM combined with 2% niacinamide, twice daily for 3 wk	● Wrinkle index ● Melanin index ● Global Aesthetic Improvement Scale (GAIS) ● Global Improvement Score (GIS) ● Safety	● Wrinkle and pigmentation significantly decrease on the ADSC-CM + niacinamide side ● Patient satisfaction reported higher for ADSC-CM + niacinamide side ( *p* < 0.05) ● No adverse effects reported from the study product
Kim, et al (2019) ^13^	Human umbilical cord blood-derived mesenchymal stem cells (hUCBMSC)-conditioned media	23	hUCBMSC-conditioned media containing cream with or without stem cell-containing serum	● Total area of microcrusts ● Erythema ● Skin biophysical parameters (corneometer, TEWL) ● Global improvement scores for skin texture	● The study group that applied both serum and cream experienced a decrease in the total area of microcrusts ( *p* < 0.05). ● The global improvement score of the post-treatment erythema was significantly reduced. ● The global improvement scores were higher in the combination treatment group
Chernoff, et al (2021) ^14^	Human placental mesenchymal stem cell-derived exosomes	40	Microneedling + topical application of 5 billion exosomes	● Skin tone ● Skin quality ● Skin clarity ● Wrinkles ● Pores ● Pigment ● Oiliness ● Improvement in the evenness of skin ● Vascularity ● Subject satisfaction	● The treatment group exhibited enhanced skin tone, quality, and clarity relative to the control group, alongside a decrease in wrinkles, pores, pigmentation, and oiliness, and improvement in skin evenness, vascularity, continuous increase in satisfaction with results from 30 to 120 days ( *p* < 0.0001)
Yusharyahya, et al (2023) ^15^	Adipose-derived mesenchymal stem cells (ADMSCs) secretome	60	Microneedling (MN) vs. fractional CO _2_ laser (FL) as methods of delivery for ADMSCs secretome in the treatment of aging skin	● Dermoscopy Photoaging Scale (DPAS) ● Wrinkles	● Significant improvements in total DPAS ( *p* < 0.01) and wrinkles ( *p* < 0.001) were found in the MN and FL groups at the end of the trial
Ichihashi, et al (2023) ^16^	Autologous ADSCs	8	Single intradermal injection of 1 × 10 ^8^ ADSCs	● Wrinkles ● Pore size	● Reduction and eventual elimination of wrinkles, encompassing the glabella, lower eyelids, crow's feet, forehead, and nasolabial folds, one to several months post-treatment. ● Double eyelids become prominent, and facial pores markedly diminish in size.
Charles-De-Sá et al (2015) ^17^	ADSCs	6	Subdermal injection of 2 × 10 ^6^ ADSCs	● Elastic fiber network (elastosis) ● The appearance of new oxytalan elastic fibers in papillary dermis	● Reduction in the elastic fiber network (elastosis) and the appearance of new oxytalan elastic fibers in the papillary dermis
Wei et al (2017) ^18^	Nano fat-derived stem cells (NFSCs)	139	NFSCs+ platelet-rich fibrin	● Facial soft tissue depression symptoms ● Skin texture ● Subject satisfaction	● Symptoms of facial soft tissue depression and skin texture exhibited greater improvement after nanofat transplants compared with traditional transplantation methods. ● The nanofat group reported an overall satisfaction rate exceeding 90%
El Kahky et al (2017) ^19^	ADSCs	20	Conventional lipofilling vs ADSCs for hand rejuvenation	● Prominence of veins ● Metacarpal spacing ● Aesthetic preference ● Mertz scale	● In all patients, both hands exhibited statistically significant aesthetic enhancement across all parameters and the Mertz scale ( *p* < 0.05)

**Table 5:** Stem cell therapy in cartilage and hair

Author and year of publication	Stem cell type	No. of subjects ( *n* )	Intervention in study	Outcome variable studied	Result of study
Castro-Govea et al(2023) ^20^	Micrografts enriched with adipose-derived mesenchymal stem cells (ADMSCs)	1	Fat was collected and divided into two samples, nanofat and microfat. Nanofat was used to isolate the ASCs; microfat was enriched with ASCs and used for nasal modeling	● Nasal tip	**● Innovative technique** : The study introduces “rhino cell,” a novel approach that combines lipoinjection with adipose-derived stem cells (ADSCs) for nasal reshaping, offering a versatile alternative to traditional rhinoplasty methods. ● **Enhanced outcomes** : Utilizing ADSCs in nasal modeling has been shown to improve tissue integration and survival rates, leading to more natural and enduring aesthetic results. ● **Minimally invasive approach** : The “Rhino Cell” technique provides a less invasive option compared with conventional surgical procedures, potentially reducing patient recovery time and associated risks. ● **Potential applications** : This method may be particularly beneficial for patients seeking minor nasal corrections or those who prefer nonsurgical interventions
Gupta et al (2023) ^22^	Allogeneic BMMSC product, Production of conditioned media derived from cultured bone marrow-derived mesenchymal stromal cells	40	The product was made using a 20% concentration of 10× conditioned media along with excipients. The final product was tested for physicochemical parameters, biomarkers, total protein content, and microbial limits as per our in-house specifications	● Hair trimming ● Dermatological assessment for efficacy ● Scalp imaging (Canon D70) ● Dermatological assessment for site application reaction. (local intolerance) ● Comb test ● Hair tensile tester ● Subject self-assessment questionnaire of hair and scalp ● Subject assessment for site application reaction ● Phototrichogram imaging ● Densitometer for hair thinning	**● Innovative treatment for androgenetic alopecia (AGA)** : The study introduces a novel hair serum, Trichosera, containing bone marrow-derived mesenchymal stromal cell-conditioned media. This formulation offers a promising non-surgical alternative for individuals experiencing hair loss due to AGA. ● **Significant improvement in hair parameters** : Clinical evaluations demonstrated notable enhancements in hair density (11.54% increase) and hair growth rate (18.66% increase) over a 24-wk period. Additionally, hair tensile strength improved by 41.10% at the 12-wk mark, indicating stronger and healthier hair strands. ● **Safety and tolerability** : The product was well-tolerated among participants, with no significant adverse events reported. This underscores its potential as a safe topical application for individuals seeking hair regeneration solutions

## Results

**Fig. 1:**
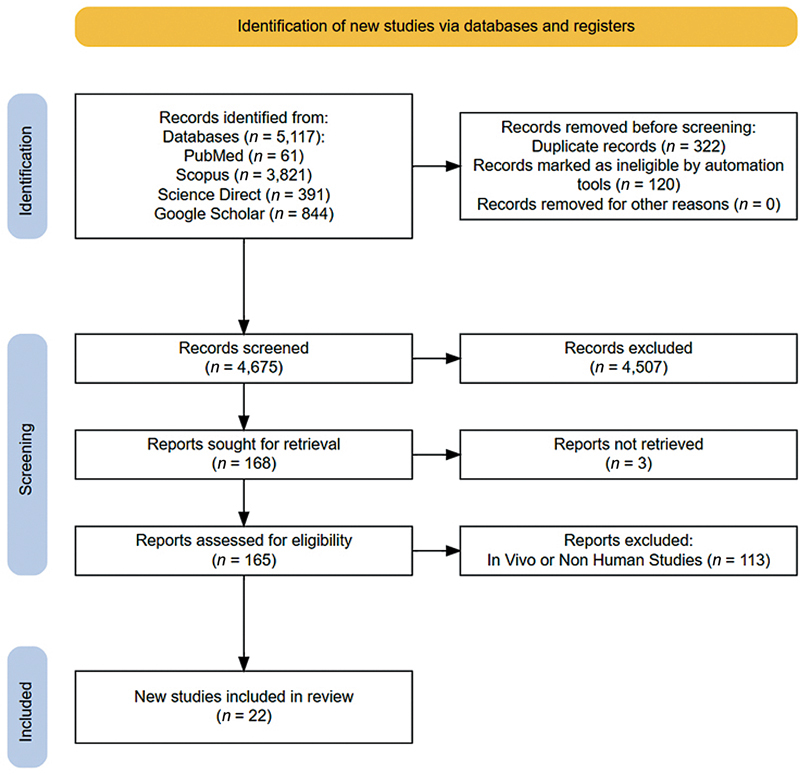
PRISMA 2020 flow diagram. 32

A total of 5,117 records were initially identified through database searches. After removing duplicates and records automatically marked as ineligible, 4,675 records remained for screening. Of these, 4,262 were excluded due to irrelevant titles, 171 studies were excluded after abstract review, and the rest were excluded as they were review articles or involved in vivo/non-human research. This leaves 22 studies that met the inclusion criteria. These 22 studies were included in the systematic review ( Fig. 1 ).

### Stem Cell Therapy in Scar Management

ADSCs and their derivatives have demonstrated notable potential in improving scar severity, enhancing skin quality, accelerating tissue regeneration, and reducing the side effects of traditional interventions like fractional CO _2_ laser resurfacing (FxCR). These findings underscore the versatility and promise of ADSCs in scar management.

Bioactive growth factors and cytokines derived from ADSC and its derivatives have been shown to enhance the effects of fractional CO _2_ laser resurfacing (FxCR) in treating atrophic acne scars and skin rejuvenation. 1 The study shows ADSC and ADSCEs have been shown to significantly improve outcomes detailed in Table 1 . 2 3 This efficiency is particularly advantageous in clinical settings where downtime and procedural frequency are key considerations.

### Stem Cell Therapy in Wound Management

On the topic of wound healing, stem cells and their exosomes play a pivotal role. They modulate inflammation, promote angiogenesis, and stimulate collagen synthesis, all essential for effective wound repair. Table 2 summarizes the studies recorded in this review.

ADSCs and ADSCEs have shown their benefit with exosomes delivering therapeutic benefits without the challenges associated with live cell therapies. 4 5

### Stem Cell Therapy in Burn Management

Despite improvements in the management of burn injuries, chronic consequences such as pain, pruritus, joint contractures, and cosmetic concerns due to hypertrophic scars (HTSs) persist as significant challenges requiring urgent intervention. The use of human stem cell-conditioned medium (HSCM) shows promise to address said issues.

The richness of growth factors and extracellular matrix (ECM) in HSCMs contributes to skin flexibility, as well as antioxidants and cytokines that inhibit cellular death. Joo et al evaluated the effect of combination therapy using nonablative laser and HSCM on tissue regeneration following burn-induced hypertrophic scar formation shown in Table 3 . 6

### Stem Cell Therapy in Skin Rejuvenation

Stem cell therapy offers a promising, safe, and efficient way to regenerate skin, potentially leading to a breakthrough in regenerative medicine and reconstructive surgery.

Zhou et al were able to improve skin regeneration by using mechanical skin expansion in conjunction with autologous bone marrow mononuclear cells (BM-MNCs) harvested from the anterior iliac crest (2.4–12.5 × 10 ^7^ cells). Histological examination showed that the dermis and epidermis were thicker and produced more collagen, giving the newly generated tissue both volume and structural integrity. 7 Afif et al highlighted that MSCs derived from the placenta are especially effective due to their high growth factor content and immune-modulatory properties. 8 Other methods of stem cell delivery shown in other studies also exhibit promising results; some authors combine human ADSCEs containing solution and microneedling, 9 10 11 while others, such as Young in Lee et al, demonstrate the efficacy of human ADSC-CM in combination with topical niacinamide. 12 The outcomes are detailed in Table 4 . 13 14 15 16 17 18 19

### Stem Cell Therapy in Cartilage and Hair

ADSCs in plastic surgery and anti-aging medicine show promise in nonsurgical procedures like nasal modeling, improving facial contours, correcting asymmetry, and enhancing tissue regeneration. Key findings are summarized in Table 5 .

While it is not a substitute for surgical rhinoplasty, ADSC is safe, reproducible, and can be repeated in less than an hour. 20 It is particularly ideal for individuals seeking less invasive options. 20 21 In this review, ADSC-CMs were primarily used for treating androgenetic alopecia. Delivery methods included microneedling and intradermal injection, often performed in multiple sessions. These therapies improved hair density, shaft thickness, and scalp vascularization, with outcomes comparable to or better than existing treatments like PRP or minoxidil. 22

## Discussion

### Stem Cell Therapy in Scar Management

This review highlights the potential of stem cell-based therapies, particularly in acne scar management, where ADSCs, ADSC-CM, and ADSCEs are used. The findings indicate that these therapies improve scar remodeling by reducing fibrosis, promoting angiogenesis, and enhancing collagen organization. 23 While individual studies vary in methodology and delivery methods, the underlying regenerative mechanisms are largely shared, emphasizing the paracrine effects of stem-cell-secreted factors.

Compared with earlier treatments such as laser resurfacing or dermabrasion, which primarily target surface remodeling, stem cell-based interventions offer a more comprehensive regenerative effect by addressing the underlying cellular environment. This review reinforces and expands on these findings by highlighting the breadth of applications and biological mechanisms involved in these therapies.

What sets cell-free approaches such as ASCEs and ADSC-CM apart is their practicality and safety profile. Without the risks associated with live cell transplantation, these products offer scalable, minimally invasive alternatives for scar management. Further economic evaluations are necessary to determine the long-term financial implications of the widespread adoption of these therapies in scar management.

Despite promising results, most studies to date are small, with short follow-up durations and non-standardized outcome measures. This makes it difficult to establish long-term efficacy or recommend a specific product or protocol. Moving forward, well-powered, comparative clinical trials with uniform endpoints are necessary to optimize the use of stem cell-derived therapies in scar treatment and validate their integration into routine aesthetic practice.

### Stem Cell Therapy in Wound Management

This review highlights the role of ADSCs and pharyngeal-derived exosomes in promoting wound healing through their ability to accelerate re-epithelialization, enhance collagen synthesis, modulate inflammatory responses, angiogenesis, and reduce oxidative stress. The findings of this review are consistent with existing literature highlighting the regenerative potential of ADSCs and exosomes in wound healing. 23

One of the major advantages of exosomes over traditional stem cell therapy is their cost-effectiveness. Exosomes retain key bioactive molecules while avoiding the logistical and regulatory challenges of live cell therapies, positioning them as a scalable and safer option in clinical wound management. Their stability and ease of application may facilitate broader use, particularly in outpatient or resource-limited settings.

Most current evidence comes from early-phase trials with small sample sizes, variable protocols, and limited follow-up. Differences in administration methods and outcome measurements further complicate direct comparison. For these therapies to move from experimental to standard care, larger randomized controlled trials with uniform clinical endpoints are essential.

### Stem Cell Therapy in Burn Management

The results underscore the need for integrating innovative therapies like HSCM into clinical practice for burn scar management. While previous studies have primarily focused on surgical and pharmacological interventions, the promising outcomes associated with HSCM introduce a novel, less invasive approach. 24 The combination of non-ablative fractional laser therapy with stem cell-conditioned media appears particularly promising. This dual approach leverages the controlled injury stimulus of laser resurfacing with the regenerative cues of stem-cell secretomes, yielding superior improvements in scar pliability, texture, and pigmentation compared with either modality alone.

While encouraging, these findings are largely derived from small studies with limited follow-up. Standardizing the timing, dosage, and frequency of these combination treatments remains a challenge. Future work should focus on optimizing protocols and confirming long-term efficacy in diverse burn populations.

### Stem Cell Therapy in Skin Rejuvenation

The results of the current review highlight the promising potential of various stem cell therapies in skin regeneration and rejuvenation. The findings from the current review align with the broader scientific consensus regarding the regenerative potential of stem cell therapies. 25 Existing literature also supports the efficacy of stem cell-derived products in stimulating collagen production, reducing oxidative stress, and promoting angiogenesis. 25 Notably, the significant improvements in skin hydration and reduction in pigmentation observed in this review are consistent with previous reports highlighting the benefits of stem cell metabolites in enhancing skin barrier function and promoting skin cell turnover.

Stem cell therapies for skin regeneration are generally more expensive than conventional skincare treatments due to the complex processes involved in cell cultivation, storage, and application. The cost may vary depending on the type of stem cell used, the delivery method, and the clinical setting. However, the significant clinical benefits often justify the higher initial costs. Many patients report high satisfaction rates, reduced signs of aging, and longer-lasting results compared with traditional treatments. Additionally, stem cell therapies may reduce the need for repetitive treatments over time, potentially lowering long-term skincare expenses.

Despite the promising results, several limitations must be acknowledged. Many of the studies reviewed had small sample sizes, which may limit the generalizability of the findings. The heterogeneity in study designs, including variations in stem cell sources, dosages, delivery methods, and outcome measures, makes direct comparisons challenging. Standardized protocols are needed to facilitate more reliable comparisons and systematic evaluations. The long-term effects and durability of stem cell treatments remain unclear. Although not reported in the reviewed studies, the potential for adverse effects cannot be completely ruled out. To validate their place in anti-aging medicine, future studies should focus on long-term efficacy, head-to-head comparisons with traditional interventions, and real-world patient satisfaction outcomes.

### Stem Cell Therapy in Cartilage

The article highlights the promising role of ADSCs in anti-aging applications within plastic and aesthetic surgery. ADSCs are easily accessible and harvested with minimal invasiveness, making them ideal for regenerative treatments. Overall, ADSCs offer a natural and minimally invasive alternative to traditional anti-aging procedures.

Over the past decade, research has increasingly supported the therapeutic potential of ADSCs in anti-aging and aesthetic procedures. The findings from the reviewed article align with a growing body of literature demonstrating the regenerative effects of ADSCs on skin quality, volume retention, and tissue repair. Yoshimura et al 26 introduced cell-assisted lipotransfer (CAL), which showed enhanced graft survival and long-term aesthetic improvements in facial and breast augmentation procedures. 26 Similarly, Gentile 27 reported that ADSCs significantly improved facial rejuvenation outcomes by enhancing dermal thickness and elasticity. 27 Recent studies also support ADSC applications in hair restoration, such as the work by Anderi et al, 28 showing improved hair density and follicle activity. 28

Compared with earlier techniques relying solely on mechanical fat grafting or synthetic fillers, ADSC-based therapies have shown superior biocompatibility, reduced complications, and longer-lasting results. These findings confirm the trends observed in the article, reinforcing ADSCs as a cornerstone in the evolving field of regenerative aesthetic medicine. ADSC therapies offer significant anti-aging benefits. However, these procedures can be costly, depending on the treatment and clinic. While the regenerative outcomes are promising, especially in facial and breast applications, the high financial cost and need for further clinical validation require careful consideration. Patients must weigh these factors alongside their personal goals and consult qualified professionals before pursuing ADSC-based treatments.

### Stem Cell Therapy in Hair

The study by Gupta et al 22 evaluated the efficacy of a hair serum containing 20% bone marrow-derived mesenchymal stromal cells (MSCs) conditioned medium in promoting hair growth and reducing hair loss, showing early promise in androgenetic alopecia. These treatments likely exert their effects through a combination of follicular stimulation, vascular support, and immunomodulation. These findings support the growing evidence over the past 10 years that stem cell-based therapies are effective in aesthetic treatments like hair restoration. This aligns with other research, such as Gentile 27 and Anderi et al, 28 which demonstrated that both adipose and bone marrow stem cells can enhance hair density and regrowth. Similarly, Kao et al 21 found that stem cell-conditioned medium boosted hair follicle activity. Together, these studies confirm the benefits and safety of stem cell therapies in anti-aging and hair loss treatments. However, the cost of such advanced biologic treatments is likely high due to complex production, though specific pricing data are not provided. Further studies with economic evaluations are needed to determine its overall value in clinical practice.

To determine their place alongside or beyond established therapies like PRP and minoxidil, larger comparative trials are needed. Long-term durability of hair growth and patient satisfaction will be key endpoints for future evaluation.

### Overall Cost–Benefit of Adipose-Derived Stem Cells Exosome and Adipose-Derived Stem Cells

ADSCs and their exosomes represent two distinct approaches in regenerative medicine with differing clinical and logistical implications. While ADSCs offer a broad regenerative potential due to their ability to differentiate and secrete a variety of growth factors, their use involves complex procedures including liposuction, cell isolation, expansion, and reinjection—each step requiring specialized equipment, skilled personnel, and strict regulatory oversight. This makes ADSC therapy relatively costly and less accessible for routine use. 29

In contrast, ADSC-derived exosomes are a cell-free alternative that retain key signaling molecules responsible for regenerative effects. Exosomes are easier to store, standardize, and deliver, often without the need for invasive procedures. Their off-the-shelf availability and lower immunogenicity make them attractive for widespread clinical applications. However, the current high cost of exosome purification and the lack of standardization in dosing remain barriers to broader implementation. 30 31

Although exact pricing varies by region and platform, the procedural burden and regulatory complexity of live-cell ADSC therapy suggest that, in the long term, exosome-based treatments may prove more cost-effective once scalable production is optimized. Future cost–benefit analyses and comparative trials are needed to determine which approach offers the best balance of efficacy, safety, and accessibility. 29 31

## Strengths and Limitations

This study has several strengths that enhance its clinical relevance and scientific rigor. By adhering to PRISMA 2020 guidelines, it ensures a systematic and transparent selection of literature, minimizing bias. The review comprehensively examines the clinical applications of stem cells in plastic, reconstructive, and aesthetic surgery, providing valuable insights for practitioners and researchers. Additionally, it explores a wide range of stem cell-based therapies, including ADSCs, exosomes, and stem cell-conditioned media (ADSC-CM), highlighting their comparative benefits in skin rejuvenation, scar management, and wound healing. By incorporating recent studies, this review captures the latest advancements in anti-aging medicine and regenerative techniques, offering a timely and up-to-date perspective on emerging trends. These strengths make this study a comprehensive resource for understanding the evolving role of stem cells in aesthetic and reconstructive procedures.

Despite its strengths, this review has several limitations. First, the included studies exhibited heterogeneity in methodology, sample size, and outcome measures, making direct comparisons challenging. Second, a meta-analysis was not performed, as the data were too diverse for quantitative synthesis. Third, only studies published in English and Indonesian were considered, potentially excluding relevant research in other languages. Fourth, most studies had short follow-up periods, limiting insights into the long-term effects and safety of stem cell therapy. Addressing these limitations through standardized protocols, long-term studies, and cost-effectiveness analyses will be essential for advancing stem cell applications in aesthetic and reconstructive surgery.

## Conclusion

ADSCs, exosomes, and stem cell-conditioned media (ADSC-CM) have demonstrated promising results in skin rejuvenation, scar management, wound healing, and volume restoration. Their ability to enhance collagen synthesis, skin elasticity, and tissue regeneration makes them valuable tools in regenerative medicine. However, long-term safety, standardized protocols, and cost-effectiveness analyses remain crucial for widespread clinical adoption. Future research should focus on large-scale, multicenter clinical trials to establish best practices and optimize patient outcomes.
